# The promises and perils of a free rural inter-city transportation scheme: A mixed-methods study from Northern Saskatchewan

**DOI:** 10.17269/s41997-024-00986-3

**Published:** 2025-02-05

**Authors:** Jacob Albin Korem Alhassan, Daniel Fuller, Ron Woytowich

**Affiliations:** 1 Department of Community Health and Epidemiology, College of Medicine, University of Saskatchewan, Saskatoon, SK Canada; 2 Kikinahk Friendship Centre, La Ronge, SK Canada

**Keywords:** Free transportation, Austerity, Budget cuts, Rural transportation, Health equity, Saskatchewan, Community-based participatory research (CBPR), Transport gratuit, Austérité, Coupes budgétaires, Transport rural, Équité en santé, Saskatchewan, Recherche participative à base communautaire (RPBC)

## Abstract

**Objective:**

Transportation is a critical health determinant, yet the last decade has witnessed rapid disinvestment across Canada (particularly in rural contexts) with negative health consequences. We sought to explore and describe the benefits and challenges faced in operating the first community-driven free-transportation scheme in Saskatchewan that emerged in response to widespread unavailability of public transportation due to budget cuts (austerity).

**Methods:**

We conducted a mixed-methods community-based participatory research study involving 22 interviews with bus riders and service administrators. We also performed descriptive statistics and chi-squared analyses on bus rider data (data on 1185 trips routinely collected between July 2023 and December 2023) to explore sociodemographic characteristics and trip purposes of bus riders.

**Results:**

All trips were completed by 616 community members using the free bus service between July 2023 and December 2023. Community members took an average of 5 trips (median = 2.0) with a maximum of 22 trips being taken by one community member (1.9% of all trips). Most trips were by women (53%), and older adults mostly used the free bus for medical purposes (22% of riders were older adults and 34% of these used the bus for medical reasons). Qualitatively, the bus service has increased access to care and promotes social participation and autonomy, especially for older adults. The service however faces some challenges, including funding disruptions and difficulty recruiting and retaining drivers.

**Conclusion:**

Free inter-community transportation (i.e. transportation across cities and municipalities) promotes health equity and access. In contexts without access to public transportation, governments could support community-driven initiatives through increased funding.

## Résumé

**Objectif:**

Le transport est un déterminant fondamental de la santé, mais la dernière décennie a été marquée par un désinvestissement rapide à travers le Canada (en particulier dans les contextes ruraux), avec des conséquences négatives sur la santé. Nous avons exploré les avantages et les défis rencontrés dans le fonctionnement du premier système de transport gratuit piloté par une communauté en Saskatchewan. L’autocar gratuit a émergé en réponse à l’indisponibilité des transports publics en raison des coupes budgétaires (austérité).

**Méthodes:**

Nous avons mené une étude de recherche participative communautaire à méthodes mixtes comprenant 22 entretiens avec des utilisateurs de l’autobus et des administrateurs du service. Nous avons également effectué des statistiques descriptives et des analyses du chi carré sur les données relatives aux utilisateurs de l’autocar (données sur 1185 trajets collectées entre juillet 2023 et décembre 2023) afin d’explorer les caractéristiques sociodémographiques et les objectifs des trajets des utilisateurs de l’autocar.

**Résultats:**

Les trajets ont été effectués par 616 membres de la communauté utilisant le service d’autocar gratuit entre juillet 2023 et décembre 2023. Les membres de la communauté ont effectué en moyenne 5 trajets (médiane = 2,0) avec un maximum de 22 trajets effectués par un membre de la communauté (1,9% de tous les trajets). La plupart des voyages ont été effectués par des femmes (53%). Les adultes plus âgés ont principalement utilisé l’autocar gratuit à des fins médicales (22% des usagers étaient des adultes plus âgés et 34% d’entre eux ont utilisé le bus pour des raisons médicales). Les analyses qualitatives démontrent que le service d’autocar a permis d’améliorer l’accès aux soins et de promouvoir la participation sociale et l’autonomie, en particulier pour les personnes âgées. Le service est toutefois confronté à certains défis, notamment les perturbations du financement et la difficulté de recruter et retenir les chauffeurs·euses de bus.

**Conclusion:**

Les transports intercommunautaires (c’est-à-dire des transports entre villes et municipalités) gratuits favorisent l’équité et l’accès à la santé. Dans les contextes où il n’y a pas d’accès aux transports publics, les gouvernements pourraient soutenir les initiatives communautaires en augmentant leur financement.

## Introduction

Transportation is one of the most important social determinants of health. It directly affects population health through road traffic injuries (Fuller & Morency, 2013) and healthcare access (Nieuwenhuijsen et al., 2016). Transportation systems also directly affect health by determining opportunities for physical activity. Good transportation systems affect chronic disease outcomes by reducing rates of obesity, diabetes, and heart disease because of opportunities for walking to bus stops or on sidewalks (Besser & Dannenberg, 2005). Where transportation systems are delivered mostly publicly, there are fewer vehicles on the road leading to lower levels of noise and air pollution with several salutogenic benefits for the general population (Khreis et al., 2017).

Beyond the direct connections described, transportation also influences health by affecting intermediate factors that subsequently promote or deplete health. The organization of transportation systems affects people’s ability to access and maintain jobs. Where there is an inadequate supply of transportation in neighbourhoods that are already dealing with other forms of vulnerability, there may be a spatial mismatch, worsening unemployment as people are unable to access jobs (Gobillon et al., 2007). These patterns of unemployment subsequently affect health through the stress of being unemployed or the lack of material resources associated with a reduced or insufficient income. The availability of good transportation options also promotes mental health by facilitating social interactions (Christie et al., 2017) and supporting people’s ability to access critical resources such as grocery stores for good food (Ghosh-Dastidar et al., 2014).

Where transportation systems are absent or inadequate, the associated negative health outcomes, such as high disease burdens, high road traffic injury rates, or inaccessibility to social and economic opportunities and healthcare, are felt unequally. The burdens of unavailable, inconsistent, and unreliable transportation options and their impacts on health disproportionately affect women, youth, Indigenous populations, people with disabilities, immigrants, and other equity-deserving groups (Alhassan et al., 2023). In the Canadian and Saskatchewan contexts in particular, how public transportation is organized and delivered can even determine exposure to violence for Indigenous women and girls through hitchhiking (Government of Canada, 2019). This research project set out to explore the promises and perils of a community-driven free-transportation scheme emerging in a context of widespread unavailability of public transportation due to budget cuts (austerity). Specifically, we aimed to describe the benefits of a free bus program and to identify challenges in running that service.

### Austerity politics and transportation

The last four decades have witnessed a dramatic increase in austerity policies heralded by the rise in neoliberal economic policy making. Austerity refers to “drastic but selective public expenditure cuts” (Schrecker & Bambra, 2015). Austerity policies are often guided by neoliberal doctrines that view the state as inherently inefficient while presenting the market as the best mechanism for organizing human interactions (Blyth, 2013). Neoliberalism itself emerged as a reaction to the economic crises of the 1970s that saw major criticisms of Keynesianism (Teeple, 2017). Neoliberalism became increasingly accepted because it was promoted by notable world leaders at the time, such as Margaret Thatcher, Ronald Reagan, and Helmut Kohl (Przeworski, 1995).

In the Canadian context, the rise in neoliberalism has involved widescale privatization and austerity policies that undermine the historical policy orientation of viewing health as a human right, favouring instead the individualization of the responsibility for health as epitomized in the Lalonde Report of the mid-1970s (Polzer & Power, 2016). More recent scholarship on neoliberalization in Canada has revealed how neoliberal modes of governance and practices promote market-based practices in healthcare delivery through encroachments into long-term care, the increasing casualization of labour in healthcare that produces stressed health workers, and the transformation of knowledge production into an increasingly instrumental rather than critical endeavour. More specifically, recent evidence on neoliberal austerity in Canada reveals an atmosphere characterized by public sector hiring freezes, privatization of public services, and in many cases a retreat of federal and provincial governments from vital service provision both within and outside the healthcare sector (Evans & Fanelli, 2018; Evans & Smith, 2015).

Public transportation is increasingly one of the major targets of government austerity policies, and this has led to large-scale disinvestments across the world. In several eastern European countries, for example, the 1990s saw major cuts to railways (Taylor, 2006) while in the 2010s, countries such as Greece and Portugal pursued similar policies (Anciaes & Alhassan, 2024). In Canada, where this study occurred, there has been large-scale disinvestment in both public and private transportation options. While public disinvestment has been part of a neoliberal turn, private disinvestment has been driven by market logics that have sometimes viewed transportation, particularly in small communities, as unprofitable. Public transportation in Canada is funded through a complex patchwork of sources from federal to municipal depending on the scope of transportation services provided. From the year 1987 onwards, neoliberal economic policymaking primarily through deregulation made intercity transportation a provincial jurisdiction (Government of Canada, 2002). This situation coupled with persistent provincial austerity budgets has undermined public transportation provision. That notwithstanding, the federal government often collaborates with provincial governments to provide transportation solutions, although there are several notable examples of provinces refusing such help (MacPherson, 2019). Presently, most funding for transportation projects is through competitive federal government grants whereby non-governmental organizations and local governments apply through Infrastructure Canada (Infrastructure Canada, 2011, 2024).

The closure of the Saskatchewan Transportation Company, a 70-year-old public bus service (Alhassan, 2021), and the closure of the Greyhound Bus Company (Evans, 2021) are specific examples of this trend towards disinvestment. These trends have been associated with negative health outcomes and disconnectedness, although very few rigorous academic studies have set out to understand how communities are responding to these changes. Additionally, it is unclear how successful any community responses have been in filling service gaps and the implications of this for equity. Focussing on Saskatchewan, where the loss of intercity transportation due to austerity policies has caused major access barriers, this study sought to reveal how this situation has engendered local community responses via a free-transportation scheme.

### Saskatchewan and the closure of the Saskatchewan Transportation Company

The setting for this study was the Canadian province of Saskatchewan. Saskatchewan is part of the prairie region and has a population of 1.2 million people, ranking sixth by population size among the 10 Canadian provinces. Saskatchewan’s major intercommunity travel option, the Saskatchewan Transportation Company (STC), owned and operated by the provincial government, had been a vital mobility link for over 200,000 citizens every year before its abrupt closure in 2017 as part of a provincial austerity budget. At the time of closure, the STC had a fleet of 41 buses, connecting 253 communities and travelling 2.8 million miles per year (Saskatchewan Transportation Company, 2017). The closure of STC by the provincial government—ostensibly to save $85 million—has had implications for vulnerable populations because the company facilitated healthcare access for vulnerable populations seeking access to services. For example, patients travelling for physician-prescribed treatments could obtain a medical pass (for $53.95) that enabled unlimited travel on a specified travel corridor for 30 days (Saskatchewan Transportation Company, 2010). Youth could obtain a $100 pass to travel throughout the province all summer (Millar, 2007), seniors discounts encouraged travel for social participation for many older adults in the province, and people with disabilities were more mobile due to the accessibility of the STC (Saskatchewan Transportation Company, 2017). Previous research (Alhassan et al., 2021a, 2021b) shows that the closure has limited healthcare access and increased healthcare system costs because existing transportation alternatives are sparsely distributed and inaccessible to vulnerable populations. Regarding health system costs, destroyed medications have had to be thrown away because they do not arrive on a consistent schedule the way they had when STC was operating (Alhassan et al., 2021a, 2023).

In rural Saskatchewan where this study occurred, many communities have had to develop alternative modes of travel, although this mostly consists of asking family and friends for rides. The absence of public transportation compounds existing challenges and barriers to other social determinants of health faced by those living in rural communities since many challenges previously seen as mainly urban problems (such as homelessness) are becoming common in rural areas as well (Buck-McFadyen, 2022). Additionally, the isolation of rural living, particularly for older adults (Lamanna et al., 2020), and the vast disconnected geographies that preclude timely care access have meant that the loss of public transportation compounds vulnerability for those in rural communities. A fascinating example of a community response to the loss of the STC has been the Kikinahk Friendship Centre free bus that provides universally free transportation between two communities (La Ronge and Prince Albert) 2.5 h apart. There are two intermediate stops between the two communities, with Prince Albert being a bigger centre (37,000 people) and most other surrounding communities, including the tri-community of La Ronge, being much smaller (5200 people). La Ronge has no within-town transit system and so passengers must arrive at the bus stop on their own, while in Prince Albert there is a city transit system for getting to the bus stop. The service began in October 2022 (bus rider data started to be collected from July 2023) through operational grants from the federal government. It has faced funding disruptions but ran until June 2024. This project sought to describe the impacts of the service and highlight challenges being faced in maintaining the service.

## Methods

This study was a mixed-methods–designed community-based participatory research (CBPR) study drawing on qualitative and quantitative research methods (Creswell, 2014). This type of research draws on community relationships and prioritizes the needs of community members and community-based organizations (Hacker, 2013). In December 2022, JAKA, who had previously conducted research on the loss of the STC, read a news article about the Kikinahk free bus and wrote to RW to learn more about the project and to discuss how the service could be sustained. After an online conversation between JAKA (researcher) and RW (executive director of the Kikinahk Friendship Centre), it was agreed that JAKA would work with RW to develop a research study on the impacts of the free inter-community public transportation scheme on health and equity to provide evidence both for the centre and for policy makers. The Kikinahk Friendship Centre is an Indigenous community-based organization, providing educational and social programming in La Ronge and area. JAKA worked with RW to develop research questions, apply for ethics approval from the University of Saskatchewan (BEH3927), and initiate an unfunded study on the impacts of the free bus service. DF worked with JAKA and RW to apply for and secure research funds and to analyze emerging quantitative results.

The project began with consultative meetings between JAKA and the executive director of the Kikinahk Friendship Centre (who was the initiator of the free bus service) as well as the transportation manager (who at the time coordinated rides and scheduled drivers) to define the scope of the research project and to agree on how to explore the impacts of the community-driven transportation scheme. It was agreed that both quantitative and qualitative data would be used to explore how the bus service affects quality of life and the well-being of community members. Research objectives and data collection instruments were co-developed by JAKA and RW and finetuned with DF. It was agreed that qualitative data would be generated through individual semi-structured interviews conducted by JAKA with stakeholders and that initial quantitative data would be generated through routine data collected on the beneficiaries of the free bus service by the transportation manager, to be initially analyzed by DF. Emerging results were interpreted by the researchers (JAKA and DF) and community partners (RW).

### Individual interviews

In April 2023, semi-structured in-depth interviews were conducted with 16 bus riders and 6 people involved in the administration of the bus service, including drivers and management of the free bus scheme. The first step in this process involved putting up research posters at the friendship centre so that community members could express interest in participating in the study by writing to the researchers or calling the transportation manager. Those interested were then screened to confirm their eligibility (i.e. that they were bus riders). JAKA conducted the interviews. These interviews explored the impacts of the free bus scheme on community members’ health, quality of life, and well-being. Interviews with management also explored some of the major challenges faced in running the free bus service and the major achievements of the free bus scheme. Research participants reflected a diversity of demographic characteristics (age, gender, class, First Nation status, and geography). All semi-structured interviews were conducted in person and lasted between 0.5 h and 1 h and 15 min. Consent forms were sent to participants prior to the interviews. At the start of each interview, the form was discussed with the participants, and they signed prior to the interview. At the end of each interview, participants were given a $25 gift card as an honorarium. Interviews were audio-recorded and transcribed verbatim before importation into NVivo 12© software for organization and analysis. Qualitative data analysis was performed by JAKA. This involved inductive and deductive coding followed by the combination of codes to form categories and subsequently theming (Saldaña, 2013).

### Quantitative data

Between July 2023 and December 2023, the transportation manager of the Kikinahk Free Bus service collected trip details of those riding the bus service. When passengers phoned in to book rides on the bus, they would be asked demographic questions on their gender (what is your gender?); a proxy question on whether they were an adult, a child, or an elder; their First Nation status; and a question on trip origin, purpose, and destination. We drew on these data to reveal demographic characteristics of bus riders and performed chi-squared tests to compare differences in trip purpose by age, gender, and First Nation status. We analyzed qualitative and quantitative data separately but drew on the qualitative data to contextualize the quantitative data.

## Results

In the following sections, we present descriptive statistics and chi-squared tests of who uses the free bus service and for what purpose. We also draw on the qualitative interview data to illustrate interviewees’ descriptions of how the free bus affects them and some of the challenges the free bus service management have faced in their attempts to run the bus service.

### Quantitative findings

Between July 2023 and December 2023, 1185 trips were completed by 616 community members using the free bus service. Community members took an average of 5 trips (median = 2.0) with a maximum of 22 trips being taken by one community member (1.9% of all trips). A majority of trips were completed by women and the main purpose for using the free bus service was for family visits. Table 1 presents demographic characteristics of bus users and the percentage of trips for each population demographic.

**Table 1:** Details of trips undertaken by beneficiaries of the free bus service

	Category (*n* (%))
Gender	
Woman	629 (53%)
Man	553 (47%)
Missing	3
Age group	
Adult	847 (71%)
Child	79 (7%)
Elder	259 (22%)
First Nations status	
Status	800 (68%)
Non-status	380 (32%)
Other	5
Reason	
Cultural	29 (2%)
Errands	45 (4%)
Legal	8 (1%)
Medical	258 (22%)
School	18 (2%)
Shopping	58 (5%)
Family visit	726 (61%)
Work	41 (3%)
Missing	2
Return trip	
No	963 (81%)
Yes	222 (19%)

Sociodemographic characteristics and trip purpose of bus riders.

**Fig. 1:**
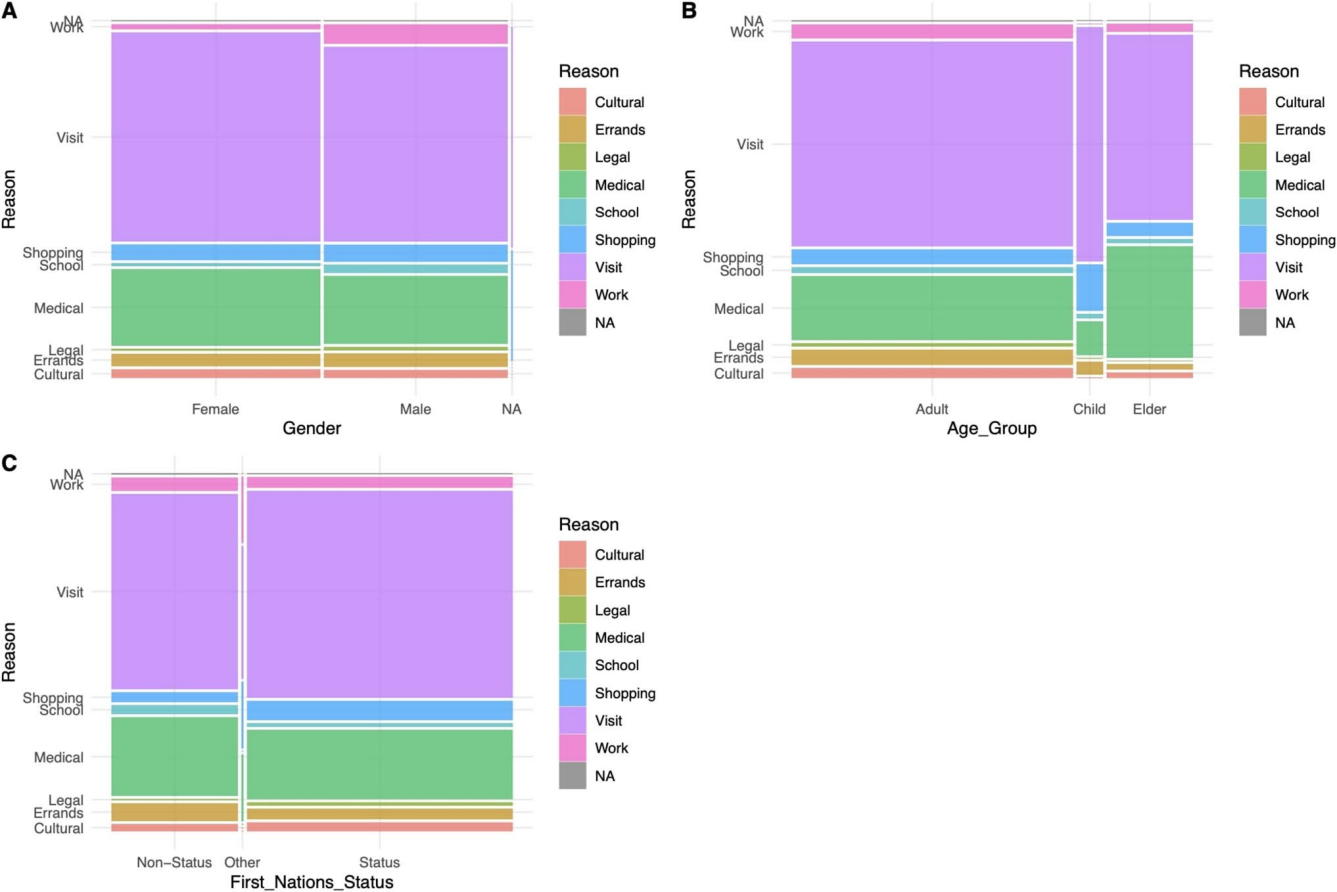
Mosaic plot of trip purpose by age group, gender, and First Nations status. **A** Reason for trip by gender. **B** Reason for trip by age group. **C** Reason for trip by First Nations status

Important sociodemographic characteristics of interest are age, gender, and First Nations status. Among those identifying as adults, a majority of the trips completed on the free bus were for family visits followed by trips for medical purposes. The group most likely to use the bus service for medical purposes were those identifying as “elders” (see Table 2). Among elders, 34% used the bus for medical reasons. Interestingly, most trips completed were one way, likely signifying the transient nature of service users or perhaps the possibility that most people combine activities during trips and do not return the same day. Men were more likely to use the service for work trips as compared with women. Elders were more likely to use the service for medical visits, while adults were more likely to use the service for shopping than both Elders and children. There were no differences in usage type of the service by status or non-status First Nations groups. Figure 1 compares percentage differences in trip purpose by age group, gender, and First Nations status.

**Table 2:** Comparing percentage differences in trip purpose by age group, gender, and First Nations status

	Cultural (*n* = 29)	Errands (*n* = 45)	Legal (*n* = 8)	Medical (*n* = 258)	School (*n* = 18)	Shopping (*n* = 58)	Family visit (*n* = 726)	Work (*n* = 41)
Age group								
Adult	25 (86)	38 (84)	8 (100)	163 (63)	14 (78)	37 (64)	525 (72)	35 (85)
Child	0 (0)	3 (7)	0 (0)	8 (3)	1 (6)	11 (19)	56 (8)	0 (0)
Elder	4 (14)	4 (9)	0 (0)	87 (34)	3 (17)	10 (17)	145 (20)	6 (15)
Significant differences	No	No	No	Elder > adult Child < adult	No	Adult > Elder Adult > child	No	No
Gender								
Woman	16 (55)	23 (51)	3 (38)	145 (56)	5 (28)	29 (51)	398 (55)	9 (22)
Man	13 (45)	22 (49)	5 (62)	113 (44)	13 (72)	28 (49)	326 (45)	32 (78)
Missing	0	0	0	0	0	1	2	0
Significant differences	No	No	No	No	No	No	No	Male > female
First Nations status								
Status	21 (72)	25 (56)	7 (88)	167 (65)	8 (44)	46 (79)	500 (69)	25 (61)
Non-status	8 (28)	20 (44)	1 (12)	90 (35)	10 (56)	11 (19)	224 (31)	15 (37)
Other	0 (0)	0 (0)	0 (0)	1 (0)	0 (0)	1 (2)	2 (0)	1 (2)
Significant differences	No	No	No	No	No	No	No	No

Chi-squared analysis comparing trip purpose of bus riders by gender, First Nations status, and age groups.

### Qualitative research findings

A total of 22 people made up of 16 bus riders and 6 people involved in the administration of the bus service participated in qualitative interviews. Bus riders were mostly women (12) while most of those involved in the administration of the bus service were men (5). Bus riders varied in age (ranging from 21 to 84).

### Benefits of the free bus service

#### Improved access to medical services

Several research participants, including First Nations communities, described the free bus service as critical in facilitating their ability to travel to access healthcare services, thereby promoting inclusion and reducing stigma for those in vulnerable circumstances. The town of La Ronge where the free bus is located is far from major hospitals and is a 6-h drive from Saskatchewan’s capital city, Regina. The closest major city (Prince Albert) for accessing groceries, hospitals, and other necessities is a 2.5-h drive south. Participants explained that having a free bus allowed them to go to cities such as Prince Albert to access care or in many cases allowed the flexibility to escort family members to hospitals. One participant noted how her grandson used the free bus to escort his mother to hospital. While the daughter attends hospital in Saskatoon, the free bus transported her to Prince Albert where she would then coordinate a subsequent vehicle to attend her medical appointment:

[My daughter] takes an injection every two weeks. [My grandson used the bus] to go help his mother or be an escort to go help his mother, my daughter. She attends medical appointments in Saskatoon [4 hours away]. And so [my grandson] would go like, tomorrow she has one in Saskatoon, right? But he used to be the escort for his mom. Then he would go to Saskatoon, and [he] has been doing that all winter. (woman, 79, free bus user #1)

Other participants described how the use of the free bus service promotes healthcare access because existing alternatives such as the medical transportation available to First Nations communities have rules that make travel difficult. For example, unlike the free bus, which is available to all community members, medical transportation for First Nations communities is only available for status First Nations people and not available for errands other than medical transportation. Additionally, a 72-h notice is often required to be able to utilize the service.

I worked for medical health services for the band. We have medical transportation that goes to PA [Prince Albert] for medical trips. It could be eyes, transplants, whatever, CAT scans, what have you. And you'd give a 72-hour notice to get on that vehicle. So, we’re finding, and I wish I had the numbers for you on how many people showed up within those 72 hours. Is it NIHB [Non-insured health benefits] Canada, that funds the medical transportation? It’s very strict, they have strict rules that you have to contact them at the office within 72 hours of the time you want to leave. Yeah, well, if you don’t get a hold of them within 72 hours, now you’re stuck, you cancel the appointment. And what if it’s a CAT scan? Or what if it’s something very important that it’s going to take another six months? (man, 60, free bus user #15)

These strict rules reveal some of the major challenges associated with alternative transportation options, hence the value of the free bus service for community members who do not have their own vehicles and must rely on other existing alternatives.

#### Improved social participation

Beyond specific benefits associated with healthcare access, several participants mentioned the service as critical for accessing health-promoting resources such as shopping for groceries but also the ability to connect with family and friends. Others described specifically how the free bus service is one of the safest travel options for visiting family and friends in the context of Saskatchewan’s harsh winters:

It was a godsend that [free] transportation, I don’t know how I was gonna get to PA [city of Prince Albert]. It’s crucial. There’s so many people. It’s a safety issue. I think that it’s a travesty if they don’t keep this bus service going…. If we’re gonna go visit, I’d be traveling with somebody to go visit probably my son. I don’t go anywhere. My mobility keeps me home. Yeah, it’s only [for] important things that I leave. I didn’t even want to come to this [interview]. I was hoping to do it over the phone. (man, 87, free bus user #6)

Several other older adults shared similar perspectives. They considered the free bus to be their main source of travel to visit family and friends and were worried about the possibility that the bus might be discontinued at some point in the future.

#### Mobility and freedom

Several participants highlighted the importance of the bus service in maintaining independence, freedom, and a sense of autonomy, particularly as people age. They highlighted how the bus service prevented them from having to seek travel favours from others. In this sense, the free bus supports dignity, allowing people who would otherwise only be able to travel thanks to others’ graciousness to not feel a sense of being beholden to others. One participant for example noted:

[Having the free bus service] means I didn’t have to beg people to travel for me. And I enjoyed the trip, right? It means that I am still independent. (man, 87, free bus user #6)

This aspect of the free bus left many people feeling that losing the bus would mean losing autonomy, freedom, and a capacity to travel. Participants often expressed that many community members found the service to be particularly useful because it is free. One participant stated how worried she was about losing the bus and why the bus is important for community members.

There’s gonna be days there’s gonna be no transportation at all for the next few months I can imagine. So, I think that’s the reason why there’s quite a few people taking advantage of this free bussing. [People say] ‘oh you’re gonna have to pay nothing? oh you’re gonna have to pay nothing?’ When you have stuff like that, that’s open to the community. I think more people take advantage of it. (woman, 52, free bus user #4)

#### Free transportation as promoter of health equity

One of the most critical themes emerging from the data is the fact that the free bus service particularly benefits vulnerable individuals and communities. Throughout the interview responses, especially in interviews with drivers and service administrators, it was made clear that the free service promotes health equity. One driver described the free bus service as follows:

It’s needed. It helps a lot of people up in the north that don’t have access to a vehicle or transportation to get to medical appointments, or even better priced groceries or shopping down south, or even for students who want to come home for the weekend, or relatives that want to go visit their children or family that are in the hospital. (man, 39, free bus driver)

In this description, the focus was on specific population subsets (including seniors, students, low-income populations, and those with existing medical conditions) enjoying improved access to care and other resources. In other interviews, when asked who the primary beneficiaries of the service are, those referenced were often elders, those experiencing homelessness, students, young families, and those without access to vehicles.

### Challenges in running the service

The free bus service faces several challenges both in terms of the resources needed to keep the service running and internal issues that have emerged from trying to operate the service. Despite the remarkable nature of the service and its uniqueness within the Canadian transportation landscape, the service does not have consistent financial support and in some cases running the service has been difficult because of lack of drivers.

#### Funding disruptions

The first major challenge in running the service is the unpredictability and insufficiency of funding. Since there is no provincially provided transportation funding, the initial source of funding had been through a combination of competitive grant applications that had not been guaranteed beyond the first couple of years of the bus service. Through interviews with the free bus program staff, it was identified many times that the free bus service may be discontinued if funding could not be secured in the future. These fears divert energy and attention away from running the free bus service and towards trying to keep the service afloat. Indeed, at some point, the entire free bus operation had to be paused because of a funding disruption. One of the program staff noted how funding challenges remain daunting:

I was told the funding ends March 31. And the next batch, if you get funding is not till May 1^st^. I have to lay the people off, because of legal [reasons], you’re gonna have to pay salaries if you didn’t give notice. So I had to give notice, which meant I had to shut the bus service down. And it’s not fair to those people, that would still be using it. (man, 67, free bus administrator)

During the funding disruption when the service ceased operation for some time while waiting for funding, one of the administrators of the service noted how anxiously he was waiting for the free bus to start up again.

There are people that are calling in [ for rides], [The free bus manager] would know. I know there are people that are calling, but I just don’t have the funding for it. But at the same time, if they [the government] called me today, if I got an email saying your funding has been approved; first, I would call our former driver and see if he wants a job and we’d start up next week! (man, 67, free bus administrator)

Despite the community demand and enthusiasm from bus management, the free bus service remained non-operational for about 3 months before further funding could be secured to start operations again.

#### Operational challenges

The management of the free bus service also described several challenges associated with running the free bus service in a small community, including difficulties in hiring and retaining drivers. Over the course of the operation of the service (2022–2024), there was high attrition—about nine drivers had worked for the service, often for short periods of time. When asked about the main operational challenges for maintaining the service, an administrator noted that they had to hire drivers and pay them more than the service could afford. Because of the limited number of buses, drivers have to be paid not only for the drive time but also for the time drivers wait to pick up the next batch of passengers, leading to drivers being paid for a 12-h day even though the return journey itself takes only about 5 h of driving. When asked the biggest operational challenges in running the service, an administrator responded:

Drivers! keeping drivers. Keeping anybody who wants to work is hard. You need a 12-hour day. Unless you got more money to run more buses, then everybody would run eight hours a day, realizing that they may get there and there’s bad weather and lots of times, they’d be coming back, and you’d be worried about them having an accident. (man, 67, free bus administrator)

Beyond the challenges of finding and keeping drivers, management of the bus service, including drivers, noted other challenges such as passengers behaving inappropriately. A bus driver described one such incident:

One mother just kept swearing at her kid. I’m a parent myself being like, Oh, you shouldn’t be yelling at your kids like that because you’re hungover and you’re coming off from your party. You know, she [the child] just wants to sing, I’m not her parent and there’s nothing I can say than to calm that woman down because it’s her child. So I just got to sit there with my mouth [shut]. (man, 39, free bus driver)

Another driver noted how winter driving can be difficult even for drivers. This situation creates some nervousness for drivers during trips.

The challenges are that in wintertime, so you have to kind of watch to see if the roads are good. If it’s a really bad day like this, no one’s really going or we’d have to go a little bit slower, take a little bit longer. The passengers didn’t mind. The passengers would get mad if the music was too loud, or they’re driving too slow. But the main thing was that everybody was safe. And everybody was taken care of. Now it’s the challenges, weather and road conditions. (man, 43, free bus driver)

These examples all highlight some of the challenges of running the bus, from the perspective of both drivers and other administrators.

## Discussion

This research project was a preliminary analysis of the health and equity impacts of a community-driven free-transportation scheme emerging in response to austerity budgets that have led to the loss of inter-community transportation options in many parts of Canada. It also described some challenges faced in running the bus scheme and the precariousness of the funding model currently being used to support the scheme. This analysis describes the first intercommunity, universally free bus service in a rural community in Canada.

Similar to extant literature on transportation and access to healthcare services (Syed et al., 2013), the scheme is critically facilitating healthcare access for the most vulnerable members of the community. Interestingly, despite this reality, most bus riders rely on the scheme to visit family and medical use is the second most common reason for use of the service. Given the critical role of social connectedness and participation in promoting health, it can still be inferred that high usage for family visits is also salutogenic.

The absence of intercommunity transportation options in Saskatchewan and much of Western Canada has serious implications not only for individuals’ health but for entire health systems (Alhassan, et al., 2021a, 2021b). Thus, while the free transportation system is serving a major community need by promoting access to care and other social determinants of health, it does not preclude the need for comprehensive publicly funded intercommunity transportation. This is because the connections between public transportation and health are complex and multifactorial, extending to environmental dimensions as well as connections to road traffic injuries (McCarthy, 2006; Nieuwenhuijsen et al., 2016). This implies that while the free bus service improves health, some population health outcomes could still become worse (e.g. road injuries) if the modal shift to using the free transportation scheme has not been sufficient to replace the proportion of people who previously used public transportation prior to the loss of the STC. This study reveals the illogic of austerity. While the STC was shut down to save money, there is no evidence that austerity saves costs—rather costs become individualized and services like the free bus we have described here may emerge to close service gaps.

Despite the remarkable ways the free bus scheme improves the well-being of individuals and communities and promotes health equity, it has a rather precarious future because of lack of financial support and perhaps insufficient understanding of its impact on community well-being. Evidence on the health benefits of free transportation in rural communities in Canada is scarce although data from suburban Ontario (Mah & Mitra, 2017) on the benefits of a free bus program (free for older adults) clearly revealed that older adults felt they would be negatively impacted by the discontinuation of the free transportation scheme.

Additionally, in the rural and small-town context as well, recruiting and retaining drivers to support the bus scheme has been challenging, hence the need for more sustainable strategies to keep the scheme continually functional. More public awareness of the scheme, financial support from governments, and quality improvements to the scheme’s operations could position the free bus service to become a model for rural communities across Canada.

## Conclusion

Extant evidence that lack of transportation disproportionately affects peripheralized and marginalized groups such as women, youth, older adults, and people with disabilities (Alhassan et al., 2023) combined with evidence that the free bus scheme is more utilized by vulnerable groups indicates that free rural transportation promotes equity. Although the higher usage of the free bus by vulnerable populations likely reveals transportation disadvantage for these groups, it also highlights the possibilities of promoting access for vulnerable populations through the free bus. While cost concerns often lead governments to disinvest in rural public transportation in response to changing population demographics (Alhassan et al., 2021a, 2021b), the evidence presented here reveals the value of investing in equity-focused transportation schemes. There are no counterfactual data to understand how the community would have coped had this scheme not emerged; however, the overwhelming evidence suggests that free rural transportation is a powerful vehicle for ensuring access to care, improving social participation, and supporting the mobility rights of some of those rendered the most vulnerable by lack of intercommunity transportation.

## Contributions to knowledge

What does this study add to existing knowledge?

- Study findings reveal transportation barriers for those seeking care in rural contexts.
- Study findings describe how community-driven transportation interventions could promote health equity and the challenges faced in doing so.
- The free bus service particularly benefits those living in vulnerable circumstances and could serve as a tool for promoting equity.
- Although medical transportation exists for First Nations communities, it is not always adequate to meet care needs.

What are the key implications for public health interventions, practice, or policy?

- There is the need for comprehensive rural transportation policy to promote access to care.
- Free public transportation in rural areas can promote health equity and should be coordinated by different levels of government.

## Data Availability

De-identified quantitative data are available on reasonable request. Qualitative data are presented in the manuscript.

## Code availability

Code used in analysis is available on request.
